# Ultrasonic vocalisation rate tracks the diurnal pattern of activity in winter phenotype Djungarian hamsters (*Phodopus sungorus*)

**DOI:** 10.1007/s00360-024-01556-2

**Published:** 2024-05-11

**Authors:** Christian D. Harding, Kerry M. M. Walker, Talya D. Hackett, Annika Herwig, Stuart N. Peirson, Vladyslav V. Vyazovskiy

**Affiliations:** 1 Department of Physiology Anatomy and Genetics, University of Oxford, Oxford, UK; 2 Division of Pulmonary, Critical Care, Sleep Medicine and Physiology, University of California San Diego, San Diego, USA; 3 Sir Jules Thorn Sleep and Circadian Neuroscience Institute, University of Oxford, Oxford, UK; 4 Department of Biology, University of Oxford, Oxford, UK; 5 Institute of Neurobiology, Ulm University, Ulm, Germany; 6 Nuffield Department of Clinical Neurosciences, University of Oxford, Oxford, UK; 7 The Kavli Institute for Nanoscience Discovery, Oxford, UK

**Keywords:** Ultrasonic vocalisations, Activity rhythms, Torpor

## Abstract

Vocalisations are increasingly being recognised as an important aspect of normal rodent behaviour yet little is known of how they interact with other spontaneous behaviours such as sleep and torpor, particularly in a social setting. We obtained chronic recordings of the vocal behaviour of adult male and female Djungarian hamsters (*Phodopus sungorus*) housed under short photoperiod (8 h light, 16 h dark, square wave transitions), in different social contexts. The animals were kept in isolation or in same-sex sibling pairs, separated by a grid which allowed non-physical social interaction. On approximately 20% of days hamsters spontaneously entered torpor, a state of metabolic depression that coincides with the rest phase of many small mammal species in response to actual or predicted energy shortages. Animals produced ultrasonic vocalisations (USVs) with a peak frequency of 57 kHz in both social and asocial conditions and there was a high degree of variability in vocalisation rate between subjects. Vocalisation rate was correlated with locomotor activity across the 24-h light cycle, occurring more frequently during the dark period when the hamsters were more active and peaking around light transitions. Solitary-housed animals did not vocalise whilst torpid and animals remained in torpor despite overlapping with vocalisations in social-housing. Besides a minor decrease in peak USV frequency when isolated hamsters were re-paired with their siblings, changing social contexts did not influence vocalisation behaviour or structure. In rare instances, temporally overlapping USVs occurred when animals were socially-housed and were grouped in such a way that could indicate coordination. We did not observe broadband calls (BBCs) contemporaneous with USVs in this paradigm, corroborating their correlation with physical aggression which was absent from our experiment. Overall, we find little evidence to suggest a direct social function of hamster USVs. We conclude that understanding the effects of vocalisations on spontaneous behaviours, such as sleep and torpor, will inform experimental design of future studies, especially where the role of social interactions is investigated.

## Introduction

Activity and rest alternate in a 24-h cycle in most species and this diurnal patterning (not to be confused with diurnality, a specific diurnal pattern characterised by daytime activity) dictates the timing of many behaviours that are functionally associated with these states such as vocalisation. Due to the immobility and reduced responsivity characteristics of rest states such as sleep and torpor, which are incompatible with behaviours that involve interaction with the environment (Harding and Vyazovskiy 2023), vocalisation outside of wake is considered abnormal (e.g. somniloquy or sleep talking, Silvestri et al. 2007). Timing of vocalisation during wake is an important contextual cue used to understand its functions. Vocalisation is often associated with an animal’s active period (Walsh & Inglis 1989; Hammerschmidt et al. 1994; Catchpole and Slater 2003; Brady et al. 2023) and therefore can be sensitive to changes in zeitgebers, for example light’s effects on bird song timing (Da Silva et al. 2014; Bruni et al. 2014). Concentration of vocalisations at dawn and dusk is a common feature observed in insects, amphibians, birds, fish and mammals (Farina and Ceraulo 2017). In addition, many species are also noted to vocalise during their rest period, for instance the nocturnal singing of many diurnal birds (La 2012) and the “long call” of orangutans (*Pongo* spp) (Samson et al. 2014).

Relatively little is known of how vocalisation behaviour and sleep interact. The detrimental effects of sleep loss on cognitive and motor functioning may impact on vocalisation as indicated by changes in the quality and number of Australian magpie (*Cracticus tibicen*) vocalisations following sleep deprivation (Johnsson et al. 2022). A drive to engage in vocalisation may affect the timing of one’s own vigilance states as suggested by the sleep fragmentation associated with nocturnal vocalisation in orangutans (Samson et al. 2014). Another possibility is that vocalisations produced by conspecifics represent an arousal stimulus capable of altering one’s own vigilance state. Sleep is, by definition, a reversible state of behavioural quiescence and perceptual disengagement from which animals can be aroused (Campbell and Tobler 1984), enabling them to react to potentially beneficial (e.g. mating opportunities) or harmful (e.g. predation) situations. To achieve this, sleeping animals must be able to perceive and respond to relevant external stimuli. Exposure to environmental noise, which closely approximates sounds encountered in laboratory animal facilities, is more likely to disturb sleeping rats (*Rattus norvegicus*) in the laboratory when compared to white noise (Rabat et al. 2004; Rabat et al. 2007). This can be explained if biologically meaningful sounds result in higher arousal levels, in turn causing an increase in sleep latency in awake animals or arousal from deeper states of sleep to shallower states or wake in sleeping animals (DeJoy 1984). Sound-induced sleep disruption may therefore be particularly relevant to animals that sleep in environments enriched with biologically meaningful sounds such as social sleepers. Equally, habituation to sounds presented during sleep (Thiessen 1978; Leaton and Jordan 1978) may counteract the disruptive effects of vocalisation in social species. Conducting chronic acoustic observational investigations of the kind required to investigate such interactions has only recently been made possible by advancements in technology including passive acoustic monitoring systems in marine settings (Risch et al 2013; Leroy et al 2016) and cheap home cage analysis systems in terrestrial settings (Hobson et al. 2020).

Whether these ideas extend to other rest states has received even less attention. Torpor is a temporary physiological state characterised by reduced metabolic rate, body temperature and physical activity during periods of actual or perceived energy shortage (Heldmaier and Steinlechner 1981). Lasting anywhere from an hour to almost an entire day, it is found in many species of bird and small mammals including owls, hummingbirds, rodents, primates and bats (reviewed in Ruf and Geiser 2015). In addition to sleep-like perceptual disengagement, low body temperatures during torpor may contribute to potentially higher auditory arousal thresholds than during sleep due to the positive correlation observed between body temperature and auditory responsivity (Coats 1965; Harrison 1965). This may suggest that sound-induced torpor disruption is less likely than sound-induced sleep disruption. Nevertheless, conspecific disruption has been identified as a possible cause of torpor arousals and vocal stimuli could be involved in such events (Turner et al. 2014; Blažek et al. 2019).

The Djungarian hamster (also known as Siberian hamster) *Phodopus sungorus* is a good model for exploring questions about sleep, torpor and vocal behaviour. Djungarian hamsters exhibit spontaneous daily torpor, a phenomenon in which some species regularly enter periods of metabolic depression for < 24 h when exposed to conditions that predict energy shortages, in this case a winter-like short day photoperiod (Heldmaier and Steinlechner 1981). During spontaneous daily torpor, their body temperature may be reduced to 14 °C when maintained at an ambient temperature of 0 °C and metabolic rate reduced by 70% (Ruf and Heldmaier 1992) leading to a total energy requirement reduction of close to 40% during winter (Heldmaier and Steinlechner 1981). Daily torpor expression can be inconsistent; up to 25% of animals fail to enter torpor when maintained in a short photoperiod in the laboratory (non-responders) and those that do will not necessarily enter torpor every day (Haugg et al. 2021). Djungarian hamsters are also widely used in the field of circadian biology to investigate the roles of light cycles and the suprachiasmatic nucleus in regulating rhythms in activity, learning and memory (Ruby 2021; Ruby et al. 1996; Ruby et al. 2004, Ruby and Zucker 1992). Particular interest has been devoted to understanding why in contrast to activity and torpor timing, which retain diurnal rhythmicity throughout the year (Scherbarth and Steinlechner 2008), the amplitude of light–dark differences in the sleep–wake cycle is reduced in short day adapted Djungarian hamsters, suggesting that the influence exerted by the circadian clock on physiological and behavioural parameters is variable (Deboer et al. 2000).

In the wild, same-sex home ranges of Djungarian hamsters are non-overlapping and actively maintained by long distance communication modalities, such as olfactory cues, whilst mixed sex home ranges overlap (Wynne-Edwards 2003). In the laboratory, Djungarian hamsters cope well with social housing as demonstrated by pair-bond disruption leading to increased cortisol levels and the appearance of neuropharmacological symptoms of depression (Castro and Matt 1997). Like many other rodents, Djungarian hamsters maintained under both short day (SD) and long day (LD) conditions vocalise during social encounters in the laboratory (Keesom et al. 2015; Rendon et al. 2015). Ultrasonic vocalisations (USVs) ranging from single tones to multi-tone sequences with multi-harmonic elements are produced during a range of behaviours whilst broadband calls (BBC) are specifically correlated with aggressive behaviours (Keesom et al. 2015). Many functions have been ascribed to rodent USVs. Laboratory mice (*Mus musculus*) produce a complex repertoire of USVs in social contexts including distress calls by pups (Ehret 2005), courtship songs of males (Holy and Guo 2005; Nyby 1983) and same-sex interactions including aggression (Costantini and D’amato 2006). Stimuli inducing vocal behaviour include negative conditions (e.g. cold temperature, handling) in the case of pups (Branchi et al. 1998) and female pheromones in the case of adult males (Sipos et al. 1995). Vocalisation type and rate in mice are strongly influenced by social context. For example, pups will cease calling if placed with an unfamiliar male (Branchi et al. 1998) and mothers respond more strongly to distress calls of their own pups than alien pups (D’Amato et al. 2005). However, as in most other rodents, the spontaneous patterning of vocalisation in Djungarian hamsters outside of engineered encounters has not been reported in the laboratory or in the wild for either solitary or socially housed hamsters despite the important information this may contain about the context and therefore function of USV emittance in this species.

Here we used chronic audio and activity recordings of short day adapted animals displaying spontaneous daily torpor in both social and non-social contexts to gain insight into the vocalisations of freely behaving Djungarian hamsters. We predicted that vocalisation would be associated with the active period of nocturnal Djungarian hamsters and would be reduced during rest states including torpor. Based on previous evidence that rodent vocalisations have a social function, we predicted that socially-housed animals would vocalise more than solitary-housed animals. Finally, we predicted that hamsters would respond to conspecific vocalisation, creating the potential for interreference with rest states such as sleep and torpor in social settings.

## Methods

### Licenses, facilities and animals

All experiments were performed in accordance with the United Kingdom Animal (Scientific Procedures) Act 1986 under personal (PIL: I48133284) and project licences (PPL: PL6150427) granted by the United Kingdom Home Office and in accordance with the University of Oxford Policy on the Use of Animals for Scientific Research. Animal holding and experimentation facilities were located at the Biomedical Sciences Building (BSB), University of Oxford. This colony of Djungarian hamsters (*Phodopus sungorus*) originates from the breeding colony of Professor Herwig at Ulm University who are descendants of animals caught near Omsk, Western Siberia by Professors Figala, Hoffmann and Goldau of the Max-Planck-Institut, Germany (Hoffmann 1973). Twelve Djungarian hamsters (4 M/8F, weight = 24.1 ± 2.8 g, age = 26.7 ± 3.6 weeks, µ ± σ) were used in this study. All light cycle profiles described feature immediate square wave transitions between light and dark phases and 750 lx fluorescent room lighting. Animals were bred under a 16L:8D summer light cycle where they remained for at least 8 weeks at 21 °C during which time they were weaned into same sex-sibling cages. Cages were then moved to an experimental room where the animals were adapted to an 8L:16D winter light cycle for 14–20 weeks (16.5 ± 1.3 weeks) to induce spontaneous daily torpor prior to the experiment (Haugg et al. 2021). A shortening of the photoperiod alone is sufficient to induce torpor in Djungarian hamsters maintained at room temperature (Heldmaier and Steinlechner 1981), hence a temperature range in the experimental room similar to the breeding room was adopted (18.5–22 °C).

### Chronic monitoring experimental design

**Fig. 1:**
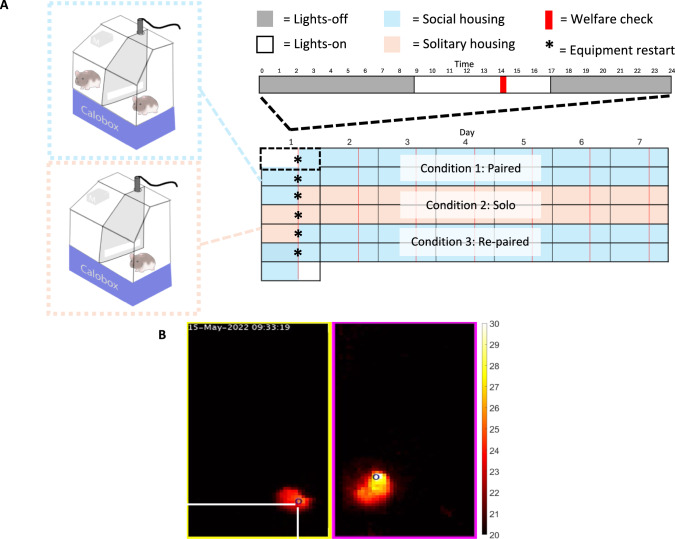
Social experiment design and equipment. **A** Hamsters were housed in separate compartments of an IVC cage divided by a metal barrier with a mesh window to allow for olfactory and auditory cue transmission and some somatosensory transmission. Thermal imaging (Optris Xi 80, cage top), indirect calorimetry (Phenosys Calobox, below cage) and ultrasonic microphone (M, Audiomoth, cage side) recordings were acquired. Animals began paired for 2 weeks followed by 2 weeks of isolation and then re-paired for a further 2 weeks. Recording equipment was restarted each week at 14:00. **B** Example thermal image showing two hamsters in a divided cage and the maximum temperature pixel used to define their position (blue circle)

To monitor torpor and vocalisation behaviour, a split-cage design similar to that used in previous rodent audio experiments (Hood et al. 2023) was implemented using a 462 mm × 403 mm × 403 mm GR1800 IVC cage (Tecniplast, Italy) divided by a solid aluminium fence in the median plane, which contained a 360 mm × 50 mm mesh window raised 40 mm above the cage floor, to create two separate compartments (Fig. 1a). The fence obstructed movement of animals between sections or from being in physical contact while recumbent, preventing social thermoregulation by huddling, but the window allowed mild physical contact whilst standing as well as the transmission of somatosensory, olfactory and auditory social cues. Each compartment contained ad libitum food and water and a limited standardised amount of bedding material (3 g kraft paper sizzle nest and 1 g cotton fibre nestlet) and wood chips (120 g) to encourage the expression of torpor and to prevent the creation of deep nests capable of concealing the infrared heat signature of the hamster. Room lighting was provided by fluorescent bulbs identical to those experienced by the animals prior to the experiment. Ventilation was provided by a Calobox indirect calorimetry system (Phenosys, Germany) set to ~ 70 L/h. Calorimetry data is not reported here as it was not possible to separate the contribution of individual hamsters to the overall gas exchange dynamics in the social housing cages. An 8-h winter photoperiod was used with illumination between 0900 to 1700 (8L:16D). Animal and food weight were measured at the start and end of the experiment.

Pairs of same-sex siblings housed together since birth were recorded in split-cages over a period of 6 weeks consisting of two periods of social-housing separated by a period of social isolation (Fig. 1a):

Condition 1: Paired—Sibling pairs were co-housed in separate compartments of the same cage for 2 weeks.

Condition 2: Solo—Sibling pairs were separated and housed in separate compartments of different cages for 2 weeks

Condition 3: Re-paired—Sibling pairs were returned to co-housed conditions for an additional 2 weeks

Fresh cages were used between conditions to eliminate transfer of olfactory cues. No cage changes occurred within conditions. Up to 3 repeats (3 × 2 animals) of the experiment were conducted simultaneously in the same 4 m^2^ experimental room using the 5 modified cages designed for this purpose with start times staggered to optimise cage occupancy time. Cage walls were translucent and although individually ventilated, shared an airspace. As a result, it is possible that hamsters were aware of the presence of other animals even in the solo condition via auditory, olfactory or visual cues transmitted between cages.

### Recording equipment and conditions

Each cage was fitted with a Xi 80 FO3 wide angle camera (Optris, Germany) which continuously captured an 80 × 80 pixel image at a sampling rate of 1 Hz suitable for detecting torpor in small mammals (Northeast et al. 2019; Huang et al. 2021). The camera was suspended from the roof along the midline to capture thermal images. The optical and temperature resolution of the camera were 12° and 0.1 K respectively. For a camera with a field of vision (FOV) of 50° and a focal length of 3.1 mm, at a measurement distance of 40 cm each square pixel should capture an area of the cage floor of 5.2 mm^2^ (https://www.optris.co.uk/optics-calculator). A similar figure of 5.7 mm^2^ was estimated using photogrammetric analysis of an image of a reference object of known dimensions placed on the cage floor using Photoshop 2022 (Adobe, USA). We therefore used an estimated pixel size of 5.5 mm^2^. The motorised focus was set to 50%. Thermal imaging was conducted continuously over the 6-week experimental period.

Audio recordings were acquired using an Audiomoth v.1.2.0 acoustic logger (Open Acoustics Devices, UK) designed for long-term monitoring of environmental sounds including ultrasonic vocalisations (Hill et al. 2019; Kunberger et al. 2023). The microphone was placed in a supporting bracket on the left side of each cage at the roof (Fig. 1a). Audio was recorded at a sampling rate of 250 kHz, facilitating the detection of vocalisations up to a frequency of 125 kHz. Due to data storage limitations, the devices were set to record on a 200/400 s active/rest alternation cycle. This resulted in a recording coverage of 1/3 of the total experiment time. A medium gain setting was used based on preliminary recordings to assess the sub–clipping threshold. Days on which > 6 h of recordings of any metric were lost were rejected from further analyses (108/516[No. animals * No. recording days]). Using only 1 microphone per cage meant vocaliser identity could only be established with certainty for solitary-housed animals. However, in the special case where only one animal in socially-housed cage was moving when a vocalisation was recorded, we presumed this animal was the vocaliser (see Results for explanation of the activity-vocalisation correlation).

Both audio and thermal recordings commenced 30 s prior to the introduction of animals to the paired condition cages on day 1 of the experiment and finished 30 s prior to removal of animals from re-paired condition cages on day 42 of the experiment. During this period, audio and thermal recordings were restarted simultaneously each week at 1400 to coincide with the relocation of animals between conditions and the daily welfare check, in which a technician entered the experimental room to view the hamsters. Audio and thermal data during the 20 min following each daily welfare check were subsequently rejected.

### Thermal image data extraction

Thermal images were saved as CSV files and analysed in the MATLAB environment (ver.R2022A, Natick, USA). The maximum temperature within the compartment occupied by each animal was chosen to represent its skin temperature (°C) and the 2D Cartesian coordinates of this pixel to represent the position of the animal (Fig. 1b). The minimum temperature in the entire image was chosen to represent the ambient temperature within the cage. Extreme temperatures > 5 standard deviations above the mean temperature in the first week, which were possible when cameras automatically re-calibrated or when the experimenter entered the camera view when opening the cage to move animals, were removed as outliers. Skin temperature and ambient temperature were mean averaged into 1-min bins then mean smoothed with a 20-min sliding window.

In a custom analysis pipeline, we compared pixel temperatures in subsequent frames to determine when animal movements occurred. When an animal moves, pixels in the previously occupied portion of the image decrease in temperature, whilst pixels in the newly occupied portion of the image increase in temperature. To remove environmental temperature changes not associated with movement, the minimum temperature for all pixels was first set to 21 °C. When the mean absolute frame-to-frame temperature change of pixels within a compartment exceeded a threshold of 0.25 °C (i.e. when subsequent images differed due to a change in the hamster’s position) a movement was scored. As there was no obvious delineation between low (unlikely to be associated with true movement) and high (likely to be associated with true movement) temperature changes, this threshold was determined by finding the elbow point of the histogram of all frame-to-frame temperature changes across the experiment**.** Whenever a movement occurred, the distance between the new position of the animal and its position when a movement was last detected was measured. Finally, to minimise the influence of welfare check disturbances on our analysis, we discarded 20 min of data following each welfare check. The presence of a movement was scored each second and binned into 1-min cumulative activity counts. Whilst our results indicate this novel method of measuring actigraphy is sensitive to diurnal changes in activity (see Fig. 3d), it has not been validated against more established methods considered to reflect ground truth and therefore caution should be taken when comparing our activity data with other studies.

### Torpor detection

Torpor was detected based on similar methods of thermal image defined torpor in mice (Huang et al. 2021) and hamsters (Ruf et al. 1991). The following method was implemented independently in each animal. First, a baseline mean and standard deviation of skin temperature during the scotophase (1700-0900), when torpor was not expected to occur, across the experiment was established. Next, we extracted all periods where skin temperature decreased to < 3 standard deviations below the baseline mean. Occasionally, temperatures would briefly drop below this threshold during periods of activity as it was possible for rearing animals to exit the camera’s field of view. Therefore, only low skin temperature periods lasting greater than 20 min were classified as a torpor bout. The entry time of each torpor bout was defined as the first time prior to the bout at which skin temperature was < 1 standard deviation below the baseline mean. Conversely the exit time of each torpor bout was defined as the last time after the bout at which skin temperature was < 1 standard deviation below the baseline mean, although this usually coincided with the daily welfare check and therefore could not be established on all days.

### Vocalisation detection and extraction

Audio WAV files were exported to the MATLAB environment (ver.R2022A, Natick, USA) for use in our custom vocalisation detection analysis pipeline inspired by elements of the VocalMat MATLAB toolbox for mouse vocalisation analysis (Fonseca et al. 2021). Each 200 s recording was divided into 1000 consecutive segments 200 ms in length and the presence of a USV was determined for each segment independently. Segments potentially containing a USV were detected on the basis of high power at high spectral frequencies in the range at which hamsters vocalise (33–91 kHz, Keesom et al. 2015). Power spectra for each segment were obtained using Welch’s method with 50 ms Hanning windows and 50% overlap (frequency resolution = 20 Hz). Segments characterised by a peak frequency > 45 kHz were scored as potential USVs and a spectrogram was generated using Kaiser windows of length 3.125 ms with 50% overlap. It should be noted that fundamental frequency, the lowest frequency in a harmonic USV, can be separate from peak frequency in rodent vocalisations (Miller and Engstrom 2010), and therefore these terms should not be treated as equivalent when interpreting our results. This threshold was set to remove segments containing common non-vocal broadband noises with a peak frequency in the ultrasound range, such as scratching. As this threshold is 14 kHz below the global mean of USV peak frequencies, 7 kHz below the mean frequency of the lowest frequency USV subtype and below the average fundamental frequency of both harmonic and non-harmonic USVs previously identified in Djungarian hamsters, we expect to capture the majority of vocalisations (Keesom et al. 2015). However, as USVs with a peak frequency as low as 33 kHz have been detected in this species, some vocalisations will be missed. Segment spectrograms were then visually inspected to remove artifacts. Specifically, segments with ultrasonic elements < 10 ms, multiple USVs or USVs that spanned multiple segments were removed from further analyses to leave only segments containing a single unbroken USV. Segments with overlapping USVs were retained only for coordination analysis (see Results: Vocalisation response analyses).

Audio segments containing a USV were filtered using a zero-phase-distortion equiripple FIR filter with a passband frequency of 40 kHz. The root mean square (RMS) envelope with a window of 2 ms was calculated for each segment and the first and last times at which the envelope exceeded a threshold of 3 standard deviations above the mean RMS of an empty cage were used to determine USV initiation time, end time and duration. A new spectrogram was generated for each filtered USV segment using the previously specified configuration. From this spectrogram the power spectra at the USV initiation and exit times were extracted. Frequency modulation coefficient of the USV was defined as the peak frequency (i.e. frequency with most power in the 45–125 kHz range) at the end of the USV subtracted by the peak frequency at the start of the USV. All vocalisation metric averages are means unless otherwise stated. USV rate was calculated per hour and multiplied by a factor of 3 (adjusted vocalisation rate) to give a more accurate estimate of the true rate after accounting for the incomplete audio sampling. USV metrics for each pair in the solo condition are formed by combining data for the separate animals.

**Fig. 2:**
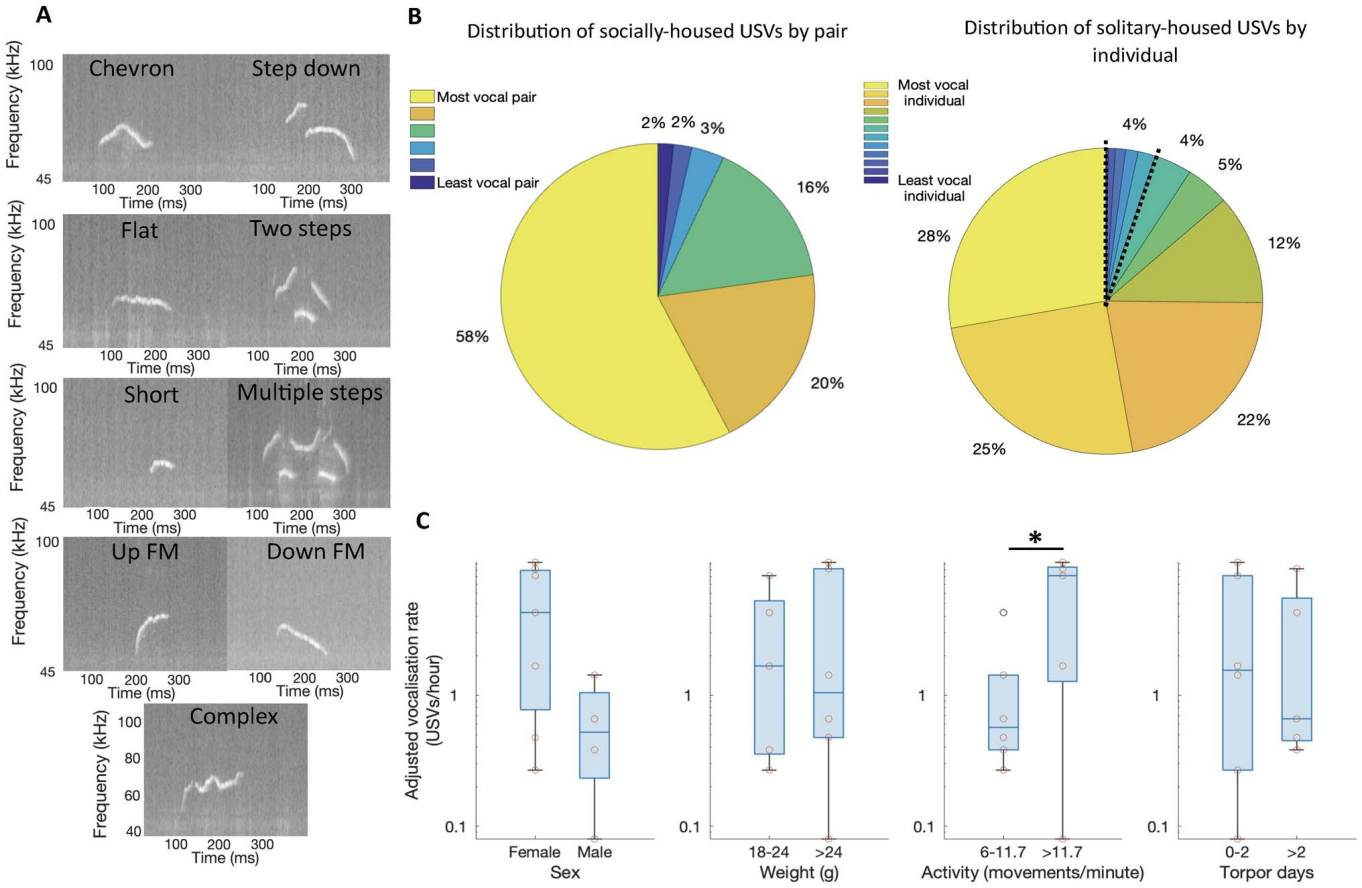
Form and prevalence of spontaneous hamster ultrasonic vocalisations. **A** Examples of the diverse spectrotemporal motifs observed in spontaneous USVs. Naming conventions derived from (Fonseca et al. 2021). Spectrograms with frequency range (40–100 kHz) chosen to highlight ultrasonic region. USVs recorded during the dark phase (1700-0900). **B** The percentage of total USVs produced by socially-housed animal pairs (top) and solitary-housed individual animals (bottom) produced by each independent sampling unit (i.e. pair or individual) is highly heterogeneous. The segment demarcated by black dashed lines (bottom) depicts the sum of the 6 least vocal individuals. **C** Boxplots depicting median, interquartile range and range in vocalisation rates of solitary-housed animals (n = 11 animals, one animal with an exceptionally low vocalisation rate was removed) grouped by sex (Female = 7 animals), weight (> 24 g = 6 animals), activity (> 11.7 movements/minute = 5 animals) and torpor duration (> 2 torpor days = 5 animals). *p < 0.05. Boxplots depict median, IQR and range

Many of the USVs recorded in this experiment suffered from mild to severe aliasing, observable in spectrograms as copies of the main USV elements repeated at higher and lower frequencies, sometimes recapitulating the pattern of frequency modulation and other times inverted. Aliasing was not a result of undersampling as the Nyquist frequency of our recording (125 kHz) was well above the maximum USV frequency of 91 kHz previously reported for this species (Keesom et al. 2015) As these aliasing elements coincide with the main USV elements temporally and are lower in amplitude, they do not influence the calculation of USV timing or peak frequency. However, they do prevent the detection of harmonic elements due to their similar appearance. As a result, we were not able to classify USVs by the presence of harmonic elements as in previous studies (Keesom et al. 2015) and therefore do no perform USV subtype analyses. However, for the benefit of future researchers, we provide examples of the different spectrotemporal motifs observed (Fig. 2a). A power threshold was applied to these spectrograms to minimise the aliased elements.

Broadband calls (BBC), the other main vocalisation type produced by Djungarian hamsters, are difficult to detect in an automated fashion as they have an overlapping frequency range with noises made by rustling in the bedding (Rendon et al. 2015). It is possible to separate these two phenomena by visual inspection of spectrograms as BBCs are louder and have a strong harmonic signature. With > 2500 h of audio, visual inspection of all recordings was not feasible for this analysis. Utilising the fact that USVs and BBCs are temporally associated in mice (Finton et al. 2017), we therefore decided to visually inspect only those recordings (200 s) which contained at least 1 USV. As none were detected, we did not investigate BBCs any further.

Temporal matching of audio derived metrics with thermal image derived metrics was limited by the time drift of these systems. Both systems were synchronized at the start of each week’s recording but as each used its own timekeeping mechanism, they drifted independently in the range of ± 4 s per day. Whilst this prevented us from accurately determining the immediate activity profile of animals at the time of vocalisation, we are able to determine the general activity level of animals around the time of vocalisation using the 1-min activity bins.

### Periodogram rhythmometry

Periodograms can be used to identify the dominant periods of a rhythmic time series. The Lomb-Scargle periodogram (Lomb 1976; Scargle 1982) is derived from Fourier spectrum analysis but can be applied to unequally sampled data and is therefore suitable for detecting rhythms in chronic observational studies where continuous data collection is challenging (Ruf 2010). A Lomb-Scargle periodogram was generated for the time series of hourly vocalisation rate using the method described by Ruf (2010) and a statistical significance threshold given a type 1 error probability of 5% was determined as follows:

$$95\% \ probability\ detection\ threshold= -ln\left[{1-(1-0.05)}^\frac{1}{N}\right]$$

where N is set equal to the number of data points. As vocalisation was rare, a single combined time series of hourly vocalisation was used to represent the overall soundscape of the experiment in periodogram analysis. The time of each vocalisation was determined in hours relative to the start of the experiment (1400 on day 0) for each animal (days 15–28) or pair of animals (days 1–14 and days 29–42) that produced the vocalisation. Vocalisation rate per hour of the experiment (total hours = 1008) was then calculated by counting the number of vocalisations produced by all animals. As the focus of this analysis was diurnal periods, periods of > 2 days were not included in the periodogram.

### Mediation analysis

We performed mediation analysis following the causal steps approach outlined by Baron and Kenny (1989). Briefly, we sought to determine whether locomotor activity (M) mediates the effect of light (X) on vocalisation rate (Y) using a sequence of linear mixed-effect models in which we treated hamster identity as a random effect to account for our repeated measure design. First, we regressed Y on X to establish that the primary predictor and the outcome variable are significantly related (effect *c*). Second, we regressed M on X to establish that the hypothesized mediator and the primary predictor are significantly related (effect *a*). Third, we regressed Y on both X and M. If the relationship between the hypothesized mediator and the outcome variable is significant even when controlling for the primary predictor (effect *b*), this suggests that M is indeed a mediator. If the relationship between the primary predictor and the outcome variable (effect *c’*) remains significant the mediation is described as partial whereas if the relationship is now non-significant the mediation is described as full. Together, the coefficients of these three models allows us to solve the equation:

$$c=c^{\prime}+a*b$$

### Vocalisation response analyses

To determine whether conspecific vocalisation contributes to arousal events in socially-housed conditions, we extracted all one-minute periods (*T*) were when one animal was active (1–60 movements) and one was inactive (0 movements) across the four weeks of social housing (paired + re-paired conditions) for each pair of hamsters. The active animal during this period was designated the actor and the inactive animal designated the spectator. These periods were categorised into 5-movement bins (no. bins = 12) based on the activity level of the actor. We then determined whether the spectator in the one-minute period immediately following each period of actor movement (*T* + 1) stayed inactive or was aroused and became active. In this paradigm, a positive correlation between the number of movements of the actor at time period *T* and the probability of arousal at time period *T* + 1 min would suggest the spectator was aroused by the actor. Finally, we divided events between those in which no USV was detected at time *T* (mute) and those in which a USV was detected at time *T* (vocal).

To determine whether conspecific vocalisation disrupted torpor in socially-housed animals, we compared the depth and duration of torpor bouts which coincided with at least one USV with the average of all torpor bouts. As a control, we also compared the activity levels of the non-torpid partner to determine whether differences in conspecific activity explained any disruption effects rather than vocalisation alone.

Overlapping pairs of USVs identified during the USV manual rejection stage that likely represent coordinated vocalisation behaviour could not be analysed spectrographically as it was not possible to separate frequency and time parameters. However, by taking the time at which ultrasonic activity first appeared to represent the overlapping vocalisation start time, we calculated the time to the nearest overlap event to assess the possibility that events were unevenly distributed in time.

### Statistics

Statistical analyses were performed in R Version 2023.06.1 (R Core Team 2023). Normality was evaluated using the Shapiro–Wilk test and non-parametric methods used if this assumption did not hold. The effect of housing condition (Levels: paired, solo, re-paired) was investigated using one-way repeated measures ANOVA (parametric) or the Friedman test (non-parametric) with post-hoc pairwise T-tests (parametric) or pairwise Wilcoxon signed rank tests (non-parametric) with Bonferroni correction for multiple testing. When the number of samples in each treatment was limited (e.g. torpor bouts), paired and re-paired treatments were concatenated and the effects of social environment (social, asocial) and light environment were investigated instead using paired T-tests (parametric) or paired Wilcoxon signed rank test (non-parametric). For thermal image temperature and activity measures, hamsters were tracked separately such that the independent unit is the animal. For audio and calorimetry recordings, we were not able to determine caller in the paired and re-paired conditions. Therefore, the independent unit in these analyses is the pair. Audio data from solo hamsters were combined to recapitulate the paired condition so that the independent unit did not change between conditions. Results are reported as mean ± standard deviation unless otherwise stated.

## Results

### What vocalisations do hamsters produce?

Contrary to previous reports (Keesom et al. 2015), both socially-housed (6/6 pairs) and solitary-housed (12/12 individuals) animals produced USVs. We did not observe broadband calls, which are known to be produced in this species in conflict settings, during the time around USV production (Keesom et al. 2015). We recorded a total of 13,113 ultrasonic vocalisations (USVs) over the course of our 6-week experiment (26 USVs/day/animal). Considering our discontinuous sampling protocol (one third coverage), the true number of USVs produced was likely in excess of 30,000 (> 60 USVs/day/animal). The repertoire of Djungarian hamster USVs was diverse. We were able to identify vocalisation elements resembling 9 of the 11 USV types characterised in a previous study of 5 strains of mixed-sex mouse pups (Fonseca et al. 2021), including chevron, flat, short, complex, down frequency modulated, up frequency modulated, short, step down, and two step motifs (Fig. 2a). Across the complete set of recordings, the peak frequency of USVs ranged from 45.0 kHz to 87.8 kHz (57.3 ± 5.2 kHz, mean ± SD peak frequency), with a mean frequency modulation coefficient of −15.8 ± 130.2 kHz/s. As the minimum peak frequency coincides with the 45 kHz threshold used for USV detection, suggesting USVs with a lower peak frequency were present but excluded, we expect that the true range of USVs produced by these animals was greater. USVs had a mean duration of 136.9 ± 49.1 ms.

Inter-pair/individual variation in vocalisation rate was considerable; 2/6 pairs contributed more than 75% of socially-housed vocalisations and 2/12 individuals contributed more than 50% of solitary-housed vocalisations (Fig. 2b). To investigate factors underlying this variation, we compared vocalisation rate across 4 binary traits in 11/12 individuals during the solitary-housed portion of the experiment (one animal was removed as an outlier due to a vocalisation rate < 1% of the mean across individuals). Activity was a significant predictor of vocalisation rate whereby animals with an above average activity level (> 11.7 movements per minute) vocalised more than animals with a below average activity level (5.9 vs 1.2 USVs per hour, T-test, *t*(9) = 2.3, *p* = 0.047, Fig. 2c). Neither weight (T-test, *t*(9) = 0.3, *p* = 0.78) nor the proportion of experiment days on which torpor was entered (T-test, *t*(9) = −0.3, *p* = 0.80) affected average hourly vocalisation rate across the 2-week isolation period (Fig. 2c). Sex also did not significantly affect vocalisation rate in solitary-housed animals however there a trend towards a higher vocalisation rate in females (4.9 vs 0.6 USVs per hour, T-test, *t*(9) = −1.94, *p* = 0.08, Fig. 2c). Indeed, despite making up only 2/3 of the study population, female hamsters were responsible for 96.5% and 93.1% of all socially-housed and solitary-housed vocalisations respectively. We suggest that a study with a balanced sex distribution and appropriate statistical power to determine sex differences may produce a different result.

### When do hamsters vocalise?

**Fig. 3:**
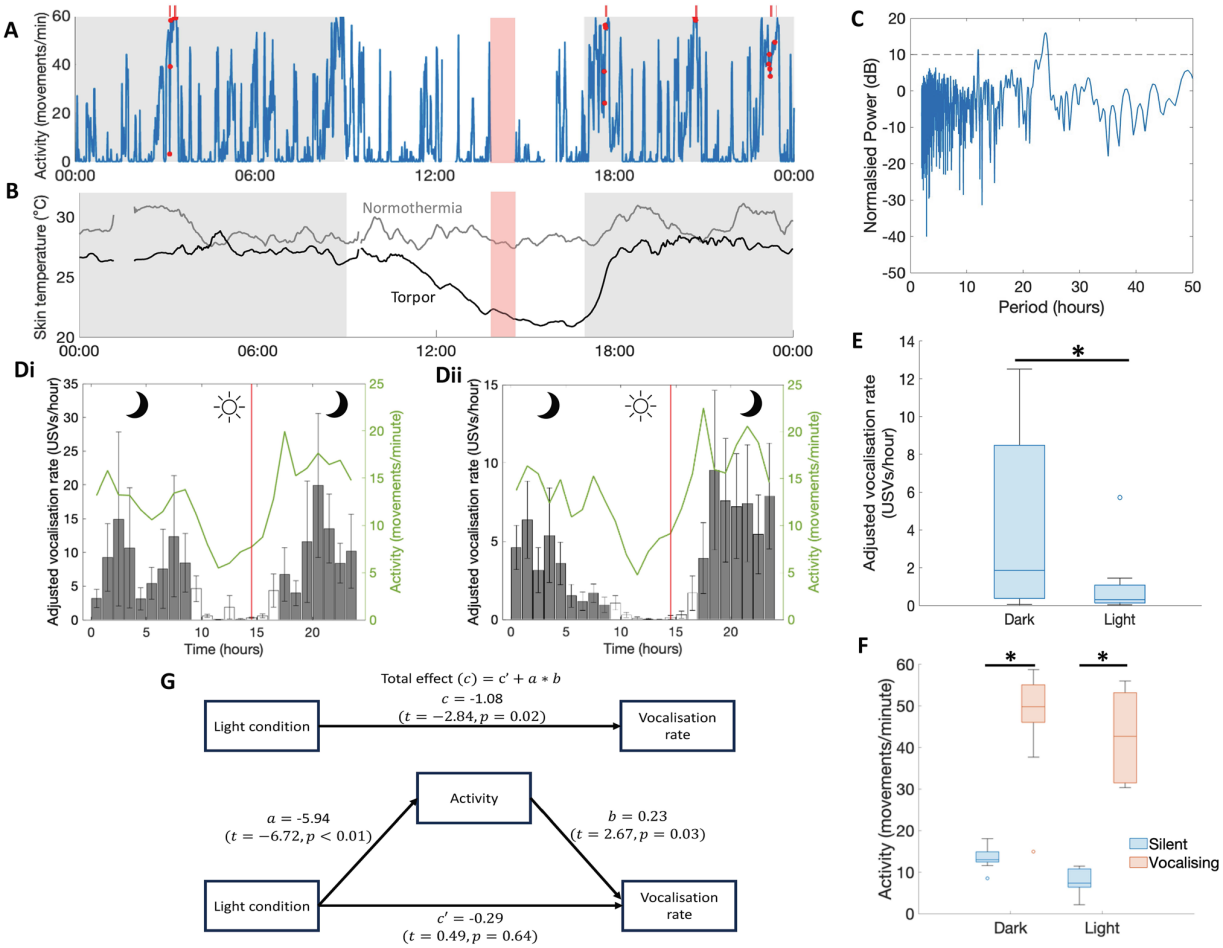
Diurnal variation in vocalisation rate associated with changes in light condition and activity. **A** Example activity profile of a solitary-housed hamster over a 24-h period. Vocalisations (red circles and bars) occur preferentially during periods of high activity. The red box denotes the period of disruption due to the daily-welfare check during which data is excluded. The lights-off period is denoted by the grey background. **B** Individual temperature profiles of a pair of socially-housed hamsters over a 24-h period. One animal entered torpor whilst the other remained normothermic. Recording breaks are the result of automatic camera calibrations. **C** Lomb-Scargle periodogram of the time-series of combined experiment vocalisations per hour with 95% probability detection threshold (dashed line). **D** Hourly vocalisation rate and activity of **i** socially-housed hamster pairs (n = 6) and **ii** solitary-housed hamsters (n = 16) is decreased during the 8 h photophase. Shading distinguishes hours where lights are on (white) or off (gray). Error bars denote SEM. The red vertical line denotes the average welfare check time (i.e. when the majority of data exclusion occurs). **E** Boxplot depicting solitary-housed hamster vocalisation rate as a function of light condition (n = 12). Significance assessed with paired T-test. **F** Boxplots depicting solitary-housed hamster activity as a function of light condition during minutes that did (red) or did not (blue) coincide with a USV (n = 12). Significance assessed with paired T-tests. **G** Schematic overview of mediation analysis to determine if activity mediates the effect of light condition on vocalisation rate (see methods). The Total Effect Model assumes that the effect of light condition on vocalisation operates directly whilst the Basic Mediation Model assumes that the effect of light condition on vocalisation rate operates through a third variable, activity. Arrows denote linear mixed-effect models. *p < 0.05. Boxplots depict median, IQR and range

One aim of this study was to characterise the temporal organisation of spontaneous vocalisation behaviour of Djungarian hamsters. Spontaneous vocalisation was rare, with whole-experiment vocalisation rate estimates ranging from 0.6–23.1 USVs per hour for socially-housed pairs and 0.03–10.3 USVs per hour for solitary-housed individuals when accounting for the intermittent recording protocol (see Fig. 3a for an example day). Animals entered torpor in all housing conditions, generally coinciding with the light phase (see Fig. 3b for an example of normothermia and torpor in a pair of socially-housed animals). Entry into and maintenance of a state of torpor appears to be incompatible with vocalisation. In single-housed conditions, in which vocaliser identity could be established with certainty, animals entered torpor on 26.8 ± 27.4% of recording days with an average duration of 2.9 ± 0.8 h, yet no contemporaneous USVs were recorded.

Hourly USV production rate in hamsters, after excluding days in which torpor occurred, displayed a clear diurnal pattern. A Lomb-Scargle periodogram of the total experiment vocalisation time series displays a dominant period of 24 h with a power of > 95% detection probability, suggesting that vocalisation rate has a 24 h rhythmic component (Fig. 3c). The diurnal modulation of vocalisation rate is characterized by a peak ~ 4 h after lights-off and reaching a nadir ~ 3 h after lights-on and is mirrored by the diurnal pattern of activity (socially-housed Fig. 3di; solitary-housed Fig. 3dii). Accordingly, mean hourly vocalisation rate in dark hours was significantly higher than during light hours for solitary-housed animals (4.2 vs 1.0 USVs per hour, paired T-test, *t*(9) = −2.8, p = 0.02, Fig. 3e). Activity rate was also significantly higher when animals were vocalising than when silent in both dark (13.5 vs 46.7, paired T-test, *t*(9) = −8.2, *p* < 0.001, Fig. 3f) and light (7.6 vs 42.5, paired T-test, *t*(9) = −9.6, *p* < 0.001, Fig. 3f) conditions, corroborating our finding that more active hamsters vocalised more frequently (Fig. 2c). In fact, less than 3% of solitary-housed vocalisations occurred when animals were immobile. This suggests that vocalisation rate is strongly associated with activity. One explanation for these findings is that because Djungarian hamsters are more active during the night in laboratory conditions, they are more likely to engage in behaviours that elicit vocalization during the dark phase. To investigate this possibility, we conducted a mediation analysis using linear mixed-effect models to determine whether activity is a mediator of the effect of light condition on vocalisation rate (see methods). We found significant effects of light condition on locomotor activity ($a=-5.9$, $t=-6.7,p<0.01$, Fig. 3g) and activity on vocalisation rate when controlling for light condition ($b=0.2,$ $t=2.7,p=0.03$, Fig. 3g), but no significant effect of light condition on vocalisation rate when controlling for activity ($c{\prime}=-0.3$, $t=0.5,p=0.64$, Fig. 3g). Locomotor activity therefore fully mediates the effect of light condition on vocalisation rate in our single-mediator model.

### Is vocalisation behaviour modulated by the social environment?

**Fig. 4:**
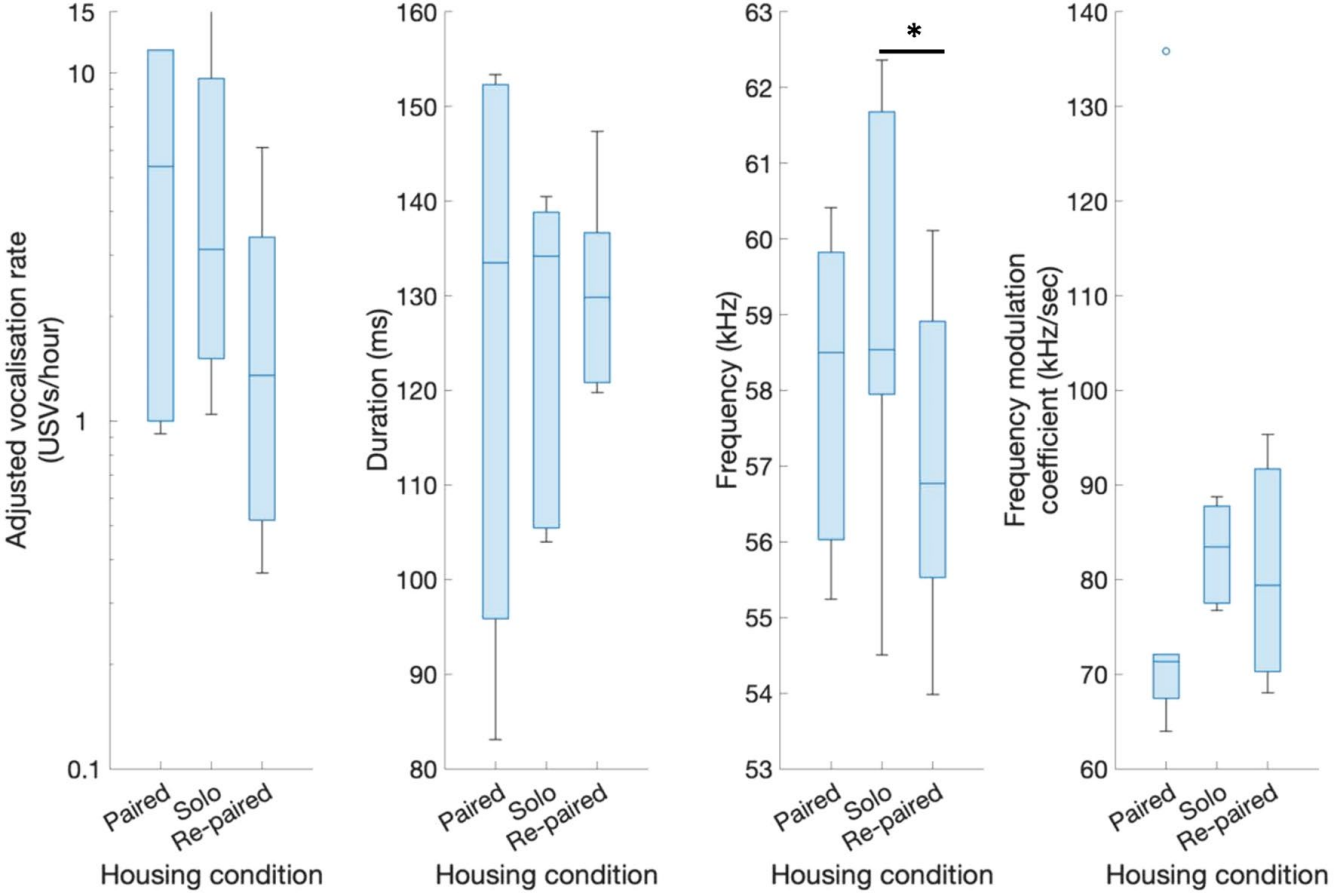
Effect of social environment on vocalisation behaviour. Same-sex sibling pairs were initially socially-housed before being separated and finally returned to their socially-housed pairings (2 weeks per treatment). Vocalisation metrics for socially-housed animals are reported for data from single cages whilst metrics for solitary-housed animals are reported for pooled data from both cages containing the initial social pair (n = 6). Social environment did not affect vocalisation rate, USV duration or the frequency modulation coefficient of USVs. In contrast, peak USV frequency was found to decrease significantly when solitary-housed animals were returned to their socially-housed pairings. Significance assessed using repeated measures ANOVA with post-hoc T-testing. *p < 0.05. Boxplots depict median, IQR and range

To determine whether social setting affected vocalisation behaviour, we performed repeated measure ANOVAs to compare pairs of animals throughout a sequence of three social settings: (1) an initial social-housed setting (paired); (2) separation of pairs into separate cages (solo); and (3) the recapitulation of initial pairings in social housing (re-paired). In the solo condition, we pooled the results from both individuals in each original pairing to allow direct comparison to the social conditions. We found no effect of social environment on USV number (*F*(2,10) = 1.6, *p* = 0.26), duration (*F*(2,10) = 0.3, *p* = 0.66) or frequency modulation coefficient (*F*(2,10) = 0.06, *p* = 0.94) (Fig. 4). There was a significant effect of social environment on the peak acoustic frequency of USVs (*F*(2,10) = 6.56, *p* < 0.05), with post-hoc multiple comparisons suggesting a minor decrease in peak frequency when solitary-housed animals were re-paired with their original partner (58.9 vs 57.0 kHz, *t*(5) = 4.9, *p* < 0.05) (Fig. 4). A significant increase in USV peak frequency was not observed when animals were originally separated (58.1 vs 58.9 kHz, *t*(5) = −1.3, *p* = 0.26), suggesting that the familiarity of animals in social housing, as opposed to social environment alone, may play a role in USV frequency.

### Do hamsters respond to conspecific vocalisations?

**Fig. 5:**
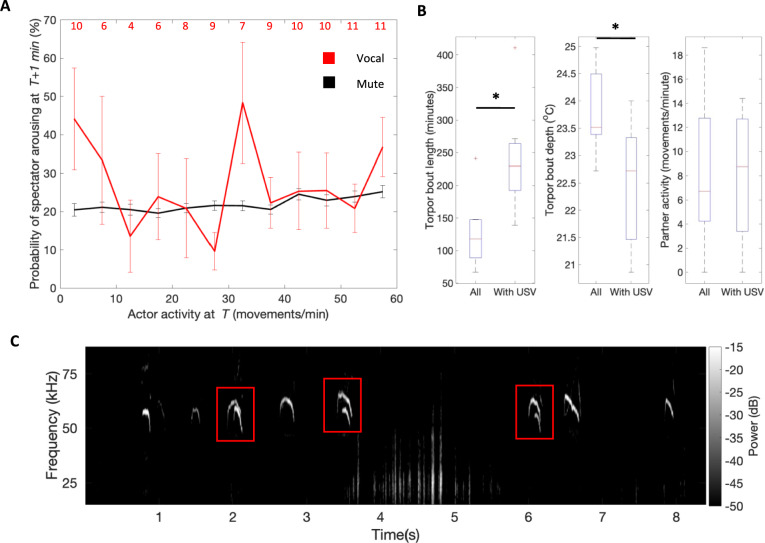
Evidence of vocalisation responses in socially-housed hamsters. **A** Probability of a ‘spectator’ (silent, immobile) cohoused with an ‘actor’ (active) at time *T* becoming active (arousing) in the following minute as a function of the actor’s activity level and whether or not the actor was vocalising at time *T*. The number of animals contributing to each vocal actor data point is displayed in red. n = 12 for all mute actor data points. Error bars depict SEM. **B** Boxplots depicting descriptive metrics of all torpor bouts in socially-housed animals or only those torpor bouts during which time a vocalisation was detected within the cage (n = 7). Both torpor bout length and depth differed depending on whether torpor coincided with a USV. Significance assessed with paired T-tests. **C** Example vocalisation train from a socially-housed hamster pair showing both overlapping and non-overlapping USVs. Red boxes denote overlapping USVs. Lower frequency activity (~ 25 kHz) most likely corresponds to movement related noise (e.g. digging, rearing against cage wall). * = *p* < 0.05. Boxplots depict median, IQR and range

Rodent USVs are known to elicit behavioural responses in mother–pup (Hofer 1996), mixed-sex adult (Willadsen et al. 2014) and same-sex adult (Brudzynski and Chiu 1995) interactions. We therefore explored the possibility that activity in socially-housed Djungarian hamsters is influenced by conspecific vocalisations. Though we were not able to distinguish vocaliser identity for socially-housed USVs in this experiment, we made the assumption that in the special case where one animal was active (> 0 movements/minute) and one was inactive (0 movements/minute), any USVs detected were produced by the active animal. This was based on the finding that the vast majority of USVs in solitary-housed animals occurred while animals were active (> 97%). To explore whether vocalisation by the active (actor) animal could influence the behaviour of the inactive (spectator) animal, we determined the probability of the spectator animals becoming active (arousing) in the minute following actor movement as a function of the actor’s activity level and whether the actor vocalised (vocal) or did not vocalise (mute) during the preceding minute. In 1/4 of instances when the actor was mute, the spectator aroused the following minute (Fig. 5a). The probability of arousal was highest when the actor was most active (25.2% at 60 movements per minute). The average arousal probability was similar when the actor was vocal (27%) but varied considerably depending on the activity level of the actor. For example, at low activity levels (5 movements per minute), average arousal probability was considerably higher when the actor was vocal (44.2%) compared to mute (20.4%).

Next, we surmised that if conspecific vocalisation is an arousing stimulus, USVs might also contribute to arousal from torpor. Socially-housed hamsters entered torpor on 22.2 ± 19.9% of days throughout the experiment, of which approximately half were interrupted by the daily welfare check. Of those that were not interrupted (i.e. those in which arousal was spontaneous), 12/63 coincided with at least one USV. Based on the finding that vocalisations never coincided with torpor bouts in solitary-housed animals, we assumed that these vocalisations were produced by the non-torpid animal. We therefore asked whether USVs in socially-housed animals showed evidence of disrupting torpor in the co-housed animal. In fact, we found the opposite relationship: torpor bouts that overlapped with a vocalisation were longer (243.0 vs 128.7 min, *t*(6) = 6.4, *p* < 0.001) and deeper (22.5 vs 23.8 °C, *t*(6) = −4.0, *p* = 0.007) than average, which could not be explained by lower partner activity (7.9 vs 8.0 movements per minute, *t*(6) = 0.0, *p* = 0.97) (Fig. 5b). Neither did the timing of vocalisations suggest an association with torpor arousal; the final vocalisation occurred closer to the start of the torpor bout than arousal in half of the overlap events (6/12). Given the infrequency of USVs in this experiment, it is possible that longer torpor bouts are more likely to coincide with a vocalisation simply by chance. However, these results could also suggest that vocalisation does not disturb torpor and may even have a stabilising effect.

Coordination is a key feature of vocal exchanges that occur in a variety of social mammal and bird species (Henry et al. 2015). The three classical types of vocal coordination are duets (overlap between 2 interlocuters), choruses (overlap between > 2 interlocuters) and antiphony (alternation between 2 interlocuters) (Henry et al. 2015). We investigated whether Djungarian hamster USVs display coordination indicative of vocal interaction. Although our lack of knowledge of vocaliser identity prevented us from detecting antiphony, we observed USVs with elements that overlapped in time and frequency on 59 occasions in socially-housed animals (only 0.7% of all socially-housed vocalisations) (for example see Fig. 5c). Based on their spectrographic structure and absence from solitary-housed animal recordings, we accredit these events to cases of simultaneous vocalisation of both hamsters. Despite their rarity, these overlap events tended to occur in bouts, with more than half coinciding within 1 min of each other. This distribution of overlap event intervals could be the result of grouping in vocal exchanges (i.e. duets). However, it may also be produced by chance during periods of intense vocalisation by both animals independent of each other, which we cannot account for in this analysis due to unidentifiable vocalisers.

## Discussion

In this study we found that winter-adapted Djungarian hamsters produce ultrasonic vocalisations spontaneously whether housed alone or in the presence of a same-sex conspecific. Spontaneous USV production varied with a 24 h period and tracked the nocturnal activity profile of these animals. In particular, USV production was lower during the 8-h light phase and did not coincide with spontaneous daily torpor events which occurred in this period. Vocalisation rate displayed high inter-subject variation but was not modulated by social context. Various forms of response to conspecific vocalisation were explored and whilst circumstantial evidence was found, we did not identify unambiguous events of vocalisation-induced arousal.

### Spontaneous vocalisation rate tracks activity

Most of the existing knowledge on the form and function of Djungarian hamster vocalisations has been acquired from experiments employing acute, artificially manufactured encounters (Keesom et al. 2015; Rendon et al. 2015). Inferences concerning the role of vocalisation in the day-to-day lives of these animals and its integration with other longer term processes such as sleep and circadian rhythms are therefore challenging to make. In the present study, we show that Djungarian hamsters produce USVs spontaneously over the course of the nychthemeron (period of 24 consecutive hours) without external input from the experimenter, both in the presence and absence of familiar conspecifics within their home cage environment. Whilst this finding contrasts previous data to suggest that Djungarian hamsters only vocalise in the physical presence of a conspecific (Keesom et al. 2015), it is consistent with evidence from other rodents that USVs are uttered even when social or sexual stimuli are absent (Musolf et al. 2010; Chabout et al. 2015; Marconi et al. 2020).

In staged same-sex intruder interactions between Djungarian hamsters, spectrotemporal properties (e.g. number of tones, harmonic elements) but not rate of production of USVs varied with both seasonal phenotype (i.e. whether or not animals were capable of spontaneous daily torpor) and sex (Rendon et al. 2015). In our chronic single housed recordings of spontaneous vocalisation, animals that entered torpor more regularly did not produce less USVs overall despite vocalisation being absent whilst in this state. This might be explained by the short duration of torpor bouts in this experiments (~ 3h) and the fact that USV rate on non-torpor days is lower at the start of the light phase when animals usually enter torpor, mitigating the relative loss of USVs. This extends the work of Rendon et al., showing that an important element of the seasonal phenotype, the occurrence in torpor, does not influence vocalisation rate. Like Rendon et al. we also did not observe a significant sex effect on vocalisation rate, however females accounted for ~ 30% more of the total vocalisation number than expected if vocalisation rate is assumed to be equal for both sexes. Sex effects on vocalisation rate are known to occur in animals such as birds, rodents and monkeys (Ballintijn and Ten Cate 1997a; Tomaszycki et al. 2001; Lenell et al. 2021; Caruso et al. 2022) but are sensitive to inter-individual variation making it difficult to detect signals in small sample sizes (Ballintijn and Ten Cate 1997b). Along with our inability to perform spectrotemporal property comparisons, a small sample size is therefore an important limitation of this study.

One factor that did affect spontaneous vocalisation rate was activity. More active animals vocalised more frequently and vocalisation rate followed a diurnal pattern in tandem with the expected activity profile of nocturnal animals. Indeed, the strong correlation between activity and vocalisation was sufficient to explain this diurnal variation independent of light conditions. The occurrence of vocalisations during the rest phase creates the possibility for interruption of sleep and torpor in social settings by the actions of conspecifics, as has been suggested by comparative and experimental studies of social species including humans and rodents (Pankhurst and Home 1984; Capellini et al. 2008; Blažek et al. 2019; Karamihalev et al. 2019). We did see a trend for low activity behaviours to be more arousing to immobile cohabitants in social-housing conditions if combined with vocalisation, however our results do not relate to sleep. Furthermore, we found no evidence of decreased torpor bout length or depth when bouts coincided with vocalisations. Thus whilst our data highlights the potential for USVs to disrupt sleep and torpor in social settings, further recordings are required to confirm any such relationship.

### Social context does not influence spontaneous vocalisation

In the current study, we did not find evidence of consistent social modulation of USVs: vocalisations occurred at a similar rate whether animals were asocially or socially housed and the only spectrotemporal change observed between social conditions was a lower peak frequency when animals were re-paired which was not observed in the original pairings. This corroborates the finding of Keesom et al., (2015) that emission of USVs was not related to aggression in Djungarian hamsters as well as evidence from mice that the presentation of same-sex olfactory stimuli has a similar effect on USV rate to a neutral stimulus such as water (Musolf et al. 2010). The most promising evidence of social modulation were the occurrences of overlapping USVs indicative of coordinated vocalisation but such was their rarity that even this may have occurred by chance during coordinated activity bouts. As such, a social modulation of vocalisation behaviour does not seems to have occurred.

There is one caveat to this conclusion; it does not hold true if hamsters perceived both the socially-housed and solitary-housed conditions as the same environment. The differences in vocalisation rate and USV spectrotemporal features between this and previous studies, as well as the absence of contemporaneous broadband calls, which are implicated in aggressive interactions, could support the hypothesis that hamsters were never aware of conspecifics in our experiment (Keesom et al. 2015). Additionally, we only used same-sex pairings to avoid the confounding effects of conflict and breeding during our chronic recordings whereas USVs in rodents are often studied in association with reproductive behavior in mixed-sex pairings (Portfors 2007). Nevertheless, the overall outcome suggested by our results is that hamsters could perceive each other in the social housing conditions. Other than direct physical touch, hamsters could see, smell and hear each other in the split-cage design. The latter is suggested theoretically by the simultaneous detection of USVs by microphones either side of the partition and empirically by the evidence of conspecific vocalisation responses in the form of a trend towards increased activity following vocalisation and the aforementioned overlapping USVs (Fig. 5). Finally, with regards to the lower vocalisation rate in this study, this only holds true when considering the average across the recording period. The maximum number of USVs observed in a 3-min recording was 76 which corresponds to an equivalent vocalisation rate of 1368 USVs/hour/pair. Thus peak spontaneous vocalisation rate in fact exceeded the rate observed in aggressive encounters (Keesom et al. 2015).

The alternate hypothesis would be that hamsters in our experiment were always aware of conspecifics. Previous evidence that Djungarian hamsters do not produce USVs in non-social contexts is based on recordings from animals housed alone in sound-attenuated chambers both in the presence and absence of conspecific olfactory cues (Keesom et al. 2015). The cages used in this experiment did not fully attenuate auditory, olfactory or visual cues meaning solitary-housed animals may still have been aware of conspecifics within the experimental room. If so, both social and solitary ultrasonic vocalisations could have served a social function in communicating territorial or mating status to perceived rivals or mates, as in mice (Portfors 2007). The primary evidence to refute this potential confound is that there was no evidence that vocalisation rate depended on conspecific proximity and as such the strength of social cues which would presumably elicit such territorial or mating behaviours.

In summary, whilst USVs may play a role in coordinating direct physical contact encounters or in mixed-sex encounters, which were not observed in this experiment, Djungarian hamsters will also spontaneously vocalise in social and non-social contexts.

### Function of spontaneous ultrasonic vocalisation

USVs have long been hypothesised as a means of social communication in rodents. Golden hamsters (*Mesocricetus auratus*; Fernández-Vargas et al. 2018), Norway rats (*Rattus norvegicus*; Bialy et al. 2016) and California mice (*Peromyscus californicus*; Pultorak et al. 2018) all demonstrate changes in the quality or quantity of vocalisations in response to changing social contexts, suggesting a role in communicating mating or aggression information to conspecifics (Fernández-Vargas et al. 2022). Such is the strength of this hypothesis in the scientific community that changes in USV rate in social interaction tests are used to measure deficits communication in mouse models of neurodevelopmental disorders such as autism spectrum disorder (Scattoni et al. 2008; Premoli et al. 2023). Recent evidence has suggested that in Djungarian hamsters USVs are an important signal used during aggressive same-sex social encounters, with differences in USV subtype use between more and less aggressive seasonal phenotypes indicating they may be involved in social communication in this species too (Rendon et al. 2015).

However it is also appreciated that social communication does not completely explain the phenomenon of USVs. For example, USVs in rats can occur both in situations with social valence like conspecific interactions (Bialy et al. 2016) and situations without social valence such as when performing memory tests (Reyes et al. 2021). In our study, spontaneous USVs occurred at a similar rate in both social and non-social contexts and occurred less frequently than reported in aggressive encounters (7 USVs/hour/pair vs 139 USVs/hour/pair) (Keesom et al. 2015). Spontaneous USVs were also spectrotemporally distinct from those produced in aggressive encounters; average duration was 50% longer and frequency modulation 100% steeper than previously reported, although peak frequency was unchanged (Keesom et al. 2015). A social communication function for the spontaneous USVs recorded in Djungarian hamsters is therefore not clear.

Another possibility is that USVs are an index of the emitter’s current affective state (Knutson et al. 2002). This may have a strong social implication, such as state of sexual arousal as suggested by evidence of USV modulation after sexual encounters (Burke et al. 2018; Marconi et al. 2020), but not necessarily. For example, various situations that have positive emotional valence can stimulate the emission of USVs in rodents, suggesting that they may simply reflect positive emotional state (Premoli et al. 2023). USVs as a marker of positive emotional state rather than sexual arousal seems more feasible in the case of winter adapted Djungarian hamsters given the regression of the gonads that accompanies seasonal acclimatization (Vitale et al. 1985). Consistent with this hypothesis, USV production was closely related to activity levels in the current study. Inter-subject variation in USV production, which is well documented in rodents (Brunelli et al. 2005; Marconi et al. 2020), may also be reconciled with this hypothesis if considerable undetected natural variation in emotional state exists in Djungarian hamster populations.

One final possibility is that these USVs are involved in echolocation. Echolocation was hypothesized as a function since the discovery of rodent ultrasonic vocalisations (Kahmann and Ostermann 1951; Anderson 1954) and was supported by early behavioural experiments in rats which demonstrated their ability to navigate mazes when blind and to discriminate distance to a platform in the dark only with intact hearing (Rosenzweig et al. 1955; Chase et al. 1980; Burn et al. 2008). This interpretation subsequently became less popular and was superseded by the communication hypothesis (Schwarting et al. 2023). However, recent confirmation of echolocation in a genus of soft-furred tree mice (*Typhlomys*) gives new credence to this possibility (He et al. 2021). Echolocation would be equally beneficial to solitary and socially housed animals which could explain the similar vocalisation rate between these groups. Vocalisations were rare and were not more common in the dark, when echolocation would be most beneficial as a replacement for vision, once activity level was taken into account. It is possible that echolocation may still be advantageous over vision when both sensory modalities are available for certain tasks such as obstacle avoidance due to better distance estimation accuracy, which may explain why other echolocating species with functional vision use echolocation when light levels are high (Eitan et al. 2022). Nevertheless, echolocation would not explain why some animals vocalise more than others unless the population had varied visual impairment which we are unable to assess. In summary a clear function of USVs is not obvious from our results, reinforcing the importance of being open to discussion when interpreting rodent USV data.

### Limitations and future studies

We identify several limitations to this investigation which must be taken into account when drawing conclusions from our results. Our inability to determine the identity of the vocaliser in socially-housed conditions severely confined the scope of our analyses of vocalisation responses. The solution to this problem is the use of microphone arrays commonly used in echolocation studies to determine the three-dimension emitter position based on time-of-arrival differences. (e.g. Gotze et al. 2016). Another limitation is our reliance on actigraphy. Actigraphy is unsuitable for sleep determination in this species as it has not been validated against gold-standard electroencephalography, hence we could only infer the relationship between the diurnal pattern of vocalisations and the activity/rest cycle and could not assess whether vocalisations represent as awakening stimulus. Aliasing in our recordings prevented us from determining the presence of harmonics or other high frequency elements in vocalisations and therefore from performing USV subtype analyses. Though our mixed-sex study population should be representative of the species, we were not powered to study sex effects on vocalisation which are known to occur in rodents and in Djungarian hamsters specifically (Caruso et al. 2022; Keesom et al. 2015). Finally, the low temporal resolution and synchronization of our audio and video recordings prevented us from determining the exact behaviour of animals during, prior to and following vocalisation, information that is vital to establishing function. For example, identifying a relationship to ‘positive’ behaviours from which can be inferred the animal’s affective state.

In addition to rectifying the aforementioned limitations, future studies should primarily focus on two key questions: (1) what is the function of hamster ultrasonic vocalisations in solitary conditions and (2) do conspecific USVs arouse hamsters from sleep? To answer the first question, chronic solitary recordings of hamster vocalisations and behaviour at a high temporal resolution in sound-attenuated chambers would be required in combination with genomic analyses. Synchronised auditory and video recordings would facilitate detection of the exact behaviours associated with hamster USV production. In addition, identifying the absence of hearing-related genes shared with other echolocating mammals would assist in ruling out echolocation as a possibility (He et al. 2021). The ideal experiment to test the second question would be a playback experiment. Previous studies have separately investigated auditory arousal thresholds from sleep using artificial low frequency sound of varying intensity (Neckelmann and Ursin 1993) and behavioural responses to USV playback focusing on waking rodents and bats (Brudzynski and Chiu 1995; Willadsen et al. 2014; Pultorak et al. 2017; Egert-Berg et al. 2018; Salles et al. 2020). Combining both forms of experiments by using playback of diverse hamster USVs at different intensities, with intensity and duration matched white noise and sham controls, to sleeping hamsters and measuring waking latency could be used to determine whether hamster USVs represent an arousing stimulus.

## Conclusion

Djungarian hamsters possess a complex repertoire of ultrasonic vocalisations that are produced during spontaneous behaviour independent of the social context. These vocalisations occur during periods of high activity and are therefore incompatible with torpor and presumably sleep. The occurrence of vocalisations in solitary animals does not preclude previous assertions that rodent USVs serve a social function but may suggest their function is more complex than currently appreciated. We found limited evidence to suggest conspecifics react to each other’s spontaneous vocalisations but given that they contain information about the state of the vocaliser (e.g. awake, active) it is possible they represent a social cue. As such, it is important to consider the role of the auditory environment in laboratory experiments with socially housed hamsters due to the possible confounding effect of their vocal behaviour.

## Data Availability

The datasets generated during and/or analysed during the current study are available from the corresponding author on reasonable request.
